# Protective Effect of Nebivolol against Oxidative Stress Induced by Aristolochic Acids in Endothelial Cells

**DOI:** 10.3390/toxins14020132

**Published:** 2022-02-10

**Authors:** Marie-Hélène Antoine, Cécile Husson, Tatiana Yankep, Souhaila Mahria, Vanessa Tagliatti, Jean-Marie Colet, Joëlle Nortier

**Affiliations:** 1 Laboratory of Experimental Nephrology, Faculty of Medicine, Université Libre de Bruxelles, Erasme Campus, 808 Route de Lennik, B-1070 Brussels, Belgium; cecile.husson@ulb.be (C.H.); tatiana.Yankep@ulb.be (T.Y.); souhaila.mahria@ulb.be (S.M.); joelle.nortier@ulb.be (J.N.); 2 Laboratory of Human Toxicology, University of Mons (UMONS), 6 Avenue du Champ de Mars, B-7000 Mons, Belgium; Vanessa.TAGLIATTI@umons.ac.be (V.T.); jean-marie.colet@umons.ac.be (J.-M.C.)

**Keywords:** aristolochic acids, nebivolol, endothelial cell, oxidative stress, metabolomic

## Abstract

Aristolochic acids (AAs) are powerful nephrotoxins that cause severe tubulointerstitial fibrosis. The biopsy-proven peritubular capillary rarefaction may worsen the progression of renal lesions via tissue hypoxia. As we previously observed the overproduction of reactive oxygen species (ROS) by cultured endothelial cells exposed to AA, we here investigated in vitro AA-induced metabolic changes by ^1^H-NMR spectroscopy on intracellular medium and cell extracts. We also tested the effects of nebivolol (NEB), a β-blocker agent exhibiting antioxidant properties. After 24 h of AA exposure, significantly reduced cell viability and intracellular ROS overproduction were observed in EAhy926 cells; both effects were counteracted by NEB pretreatment. After 48 h of exposure to AA, the most prominent metabolite changes were significant decreases in arginine, glutamate, glutamine and glutathione levels, along with a significant increase in the aspartate, glycerophosphocholine and UDP-N-acetylglucosamine contents. NEB pretreatment slightly inhibited the changes in glutathione and glycerophosphocholine. In the supernatants from exposed cells, a decrease in lactate and glutamate levels, together with an increase in glucose concentration, was found. The AA-induced reduction in glutamate was significantly inhibited by NEB. These findings confirm the involvement of oxidative stress in AA toxicity for endothelial cells and the potential benefit of NEB in preventing endothelial injury.

## 1. Introduction

Aristolochic acids (AAs) are active compounds contained in medicinal plants from the Aristolochia and Asarum species. The consumption of AAs is now recognized as a cause of iatrogenic and environmental chronic kidney disease (CKD), namely, aristolochic acid nephropathy (AAN) [1,2]. The onset and progression of renal fibrosis are experimentally characterized by an acute phase (i.e., tubular necrosis of the S3 segment of the proximal tubule reflecting acute kidney injury (AKI)) followed by marked tubular atrophy and interstitial fibrosis leading to end-stage kidney disease [3]. Beside these well-established histologic features of AAN, marked capillary rarefaction has been observed [4,5]. The reduction in vascular density is thought to promote hypoxia and could represent a predisposing factor for the progression of renal failure [6,7]. Peritubular capillary rarefaction has actually been used as a marker to evaluate the long-term impact of AKI-to-CKD progression in experimental models [8]. Among the mechanisms explaining capillary loss, the death of endothelial cells by apoptosis via the PI3K/Akt signaling pathway has been proposed [9]. Our studies have previously demonstrated that AAs induce oxidative stress in endothelial cells [10]. Excessive production of reactive oxygen species (ROS) may contribute to decreased nitric oxide (NO) bioavailability, leading to endothelial dysfunction and apoptosis or necrosis [11,12]. Moreover, Yang et al. demonstrated that the protein expression of endothelial nitric oxide synthase (eNOS) was reduced by AAs in a mouse model of AAN [13]. Restoring the bioavailability of NO by reducing oxidative stress has been shown to improve renal function in AA-treated mice [14].

Despite the limited use of AA-containing products, humans might be exposed to AAs accidentally or because of insufficient knowledge about the toxicity of plants containing AAs.

Given the lack of treatments for AAN, nebivolol (NEB), a third-generation beta-blocker drug indicated for the treatment of hypertension, may be of interest. Both in vitro and in vivo studies have shown the antioxidant potential of NEB [15,16]. NEB is a highly selective β1-blocker with the ability to stimulate eNOS, enhancing NO bioavailability [17,18]. Since capillary depletion could represent a predisposing factor for the progression of renal failure, it seems interesting to preserve vascular integrity during toxic oxidative insults.

Metabolomic technology can provide mechanistic insights into the effects of exposure to a toxic compound [19,20]. In particular, ^1^H-NMR-based metabolomics has been reported as a powerful method for studying metabolic responses during rat or human exposure to AAs [21,22]. The aim of the present study was to investigate, in vitro, the effect of NEB in human endothelial cells exposed to AA. A metabolomic approach using ^1^H-NMR was also adopted to better characterize the metabolic impact of AA in the presence and absence of NEB.

## 2. Results

### 2.1. Nebivolol Protects EAhy926 Cells against AA-Induced Cytotoxicity

**Figure 1:**
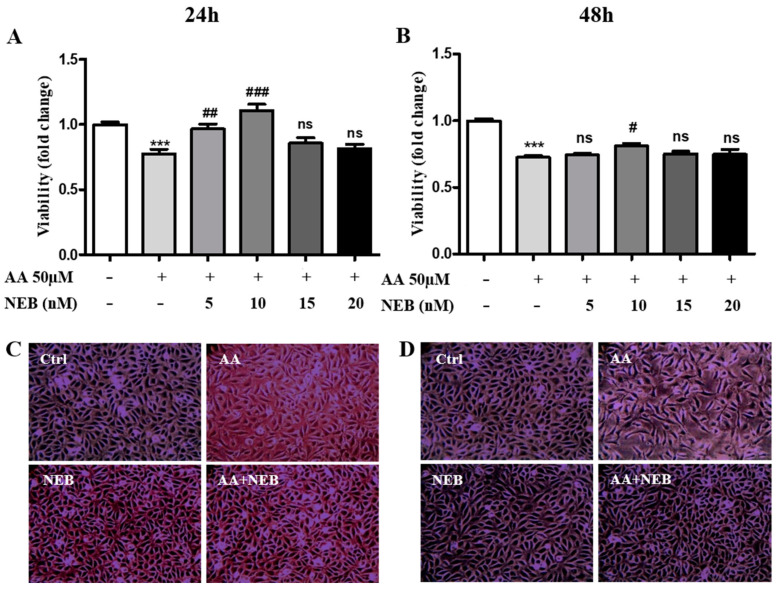
Effect of NEB on AA endothelial cells toxicity (EAhy926). Viability was determined using the resazurin test on cells cultured for 24 h (**A**) or 48 h (**B**) under standard culture conditions or in the presence of 50 µM AA and different concentrations of NEB (5, 10, 15 and 20 nM). Representative images of EAhy926 cells’ morphology under standard culture conditions or in the presence of 50 µM AA and/or 10 nM NEB for 24 h (**C**) or 48 h (**D**) (200× magnification). Values are mean ± SEM showing the fold changes in viability relative to untreated cells. Each assay was completed at least three times, with six biological replicates each. Statistical analysis was performed by one-way ANOVA followed by Bonferroni’s multiple comparison test. ns, not significant; *** *p* < 0.001 vs. controls; # *p* < 0.05, ## *p* < 0.01 and ### *p* < 0.001 vs. AA.

The cytotoxic concentration range for AA in the endothelial cell line was determined in our previous study [10]. Using the EAhy926 cell line, we confirmed that exposure to 50 µM AA for 24 h and 48 h significantly reduced the viability of EAhy926 cells compared to a control group (Figure 1A,B). In our experiments, endothelial cells were pretreated with different doses of NEB. The beneficial effect of NEB on AA cell toxicity was more pronounced at 24 h than 48 h (Figure 1A,B). Moreover, 10 nM NEB, a concentration close to the clinical plasma concentration [23], attenuated AA-induced cell toxicity more significantly compared to other doses (Figure 1A,B). We found that 10 nM NEB alone significantly increased cell viability at 24 h (data not shown). This dose was revealed to be safe as NEB toxicity by itself was detected at 150 nM (data not shown). The cell toxicity induced by AA was illustrated by phase-contrast images, showing a decrease in the number of cells and a change in the morphology of cells, which became elongated upon AA exposure (Figure 1C,D). In experimental conditions, 10 nM NEB restored the number and morphology of cells (Figure 1C,D).

### 2.2. Nebivolol Attenuates ROS Levels

**Figure 2:**
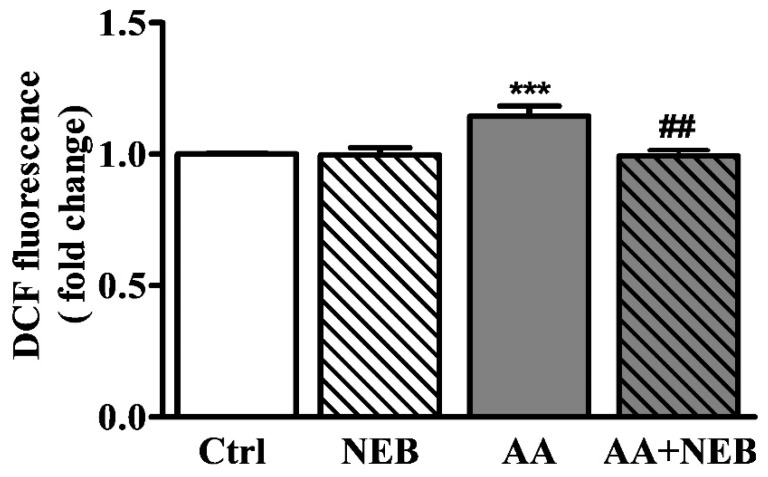
Fluorescent intensity of intracellular ROS analyzed by DCFDA in EAhy926 control cells and in cells exposed to 50 µM AA and/or 10 nM NEB for 24 h. Values are mean ± SEM showing the fold changes in fluorescence relative to untreated cells. Each assay was completed at least six times, with two biological replicates each. Statistical analysis was performed by one-way ANOVA followed by Bonferroni’s multiple comparison test. *** *p* < 0.001 vs. controls and ## *p* < 0.01 vs. AA.

The production of ROS was evaluated by the enhancement of the 2′,7′-dichlorodihydrofluorescein diacetate (DCFDA) fluorescence intensity. As shown in Figure 2, ROS production was significantly increased in cells exposed to AA for 24 h compared to controls. Importantly, preincubation with NEB significantly inhibited the increased levels of ROS induced by AA, suggesting that NEB can decrease the production of toxic free radicals.

### 2.3. Metabolism of EAhy926 Endothelial Cells under Baseline Conditions

**Figure 3:**
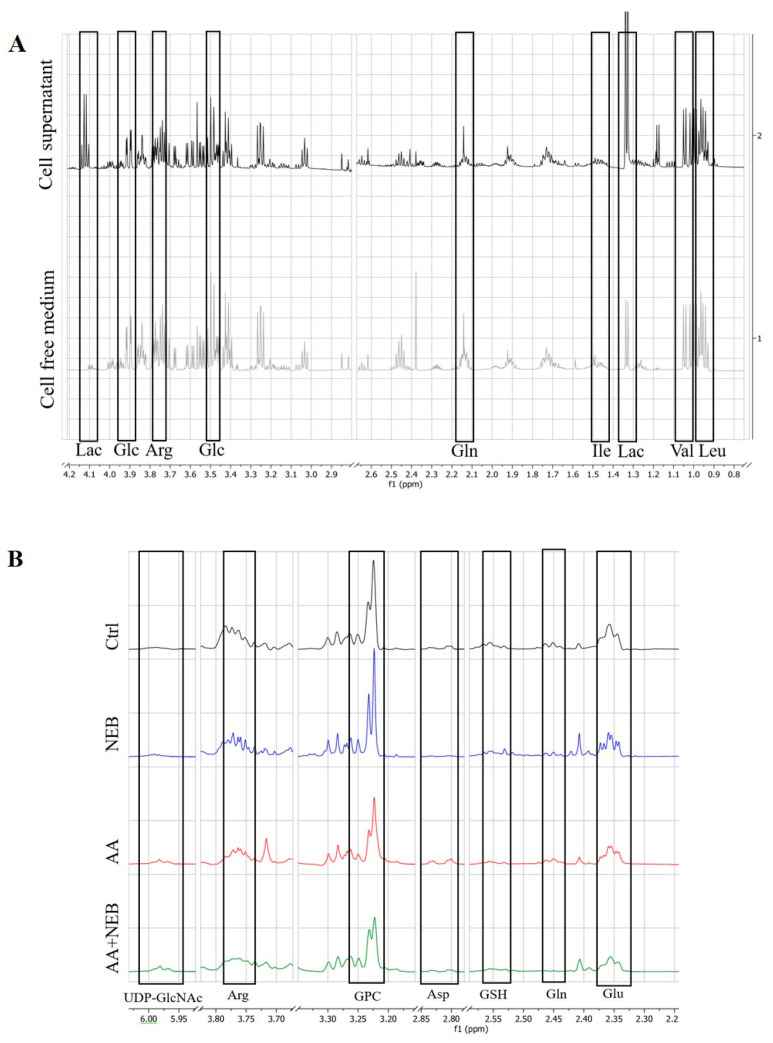
^1^H-NMR spectra (600 MHz) of cell supernatant (black) and cell free medium (grey) (**A**). ^1^H-NMR spectra (600 MHz) of intracellular extract from control group (black), 10 nM NEB group (blue), 50 µM AA group (red) and AA + NEB group (green) (**B**). Arg: arginine; Asp: aspartate; Glc: glucose; Gln: glutamine; Glu: glutamate; GPC: glycerophosphocholine; GSH: glutathione; Ile: isoleucine; Lac: lactate; Leu: leucine; UDP-GlcNAC: UDP-N-acetylglucosamine; Val: valine.

To demonstrate the basal consumption of EAhy926 endothelial cells, the supernatant from endothelial cells was compared with cell-free culture medium. A visual examination of the ^1^H-NMR spectra revealed a wide variety of metabolite resonances, which were assigned to specific metabolites according to the public NMR database (Human Metabolome Database (HMDB, Edmonton, AB, Canada,)). In the cell supernatants, a decrease in glucose and amino acids such as glutamine, valine, leucine, isoleucine and arginine, and an increase in lactate compared with the uncultured medium, were observed, reflecting the consumption of glucose and amino acids and the production of lactate by the endothelial cells (Figure 3A).

### 2.4. Intracellular Metabolic Alterations Induced by AA Exposure

Afterward, we performed ^1^H-NMR spectroscopy on aqueous-phase endothelial cell extract incubated with and without 50 µM AA and/or 10 nM NEB for 48 h to assess the global metabolic impact of each experimental intervention. Analysis of the NMR spectra of the cell extract highlighted the variety of metabolites identified by HMDB (Figure 3B).

**Figure 4:**
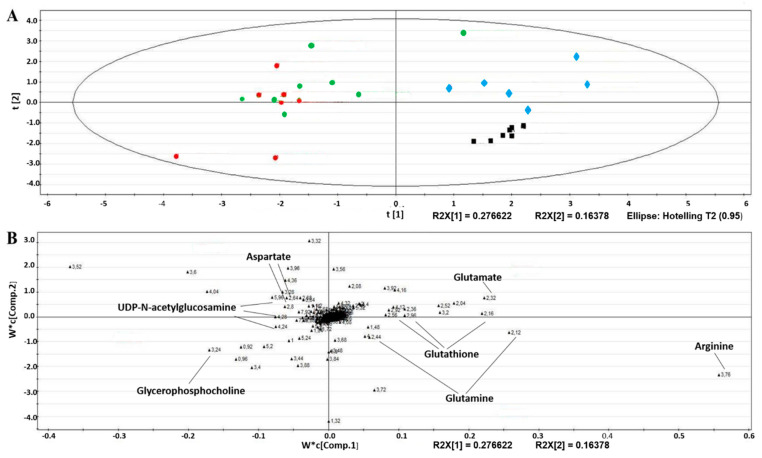
Score plots (**A**) and corresponding loading plots (**B**) of the partial least-square (PLS-DA) analysis based on the ^1^H-NMR spectra of endothelial cellular extracts from the control (black), 10 nM NEB (blue), 50 µM AA (red) and AA + NEB (green) groups.

For a comprehensive observation of the metabolic profiles from the four groups and to maximize the distinction between groups, multiple data analyses were performed on the respective NMR data for the cell extract. The score plot obtained from PLS-DA revealed a clear discrimination between the metabolomic profiles obtained from both AA-treated groups (Figure 4A to the left, the AA group (in red) and the AA/NEB group (in green)) and the groups not exposed to AA (to the right, the control group (CTRL in black) and the NEB group (in blue)). A clear separation between the CTRL and NEB groups was also observed. The discrimination between the AA and NEB/AA groups was less obvious. The significant discriminant metabolites were identified from their corresponding descriptors in the loading plots (Figure 4B). Only metabolites characterized by VIP scores higher than one were considered. The corresponding loading plots highlighted metabolites related to oxidative stress such as glutathione, glutamine, glutamate, arginine, glycerophosphocholine, UDP-N-acetylglucosamine and aspartate.

The metabolic changes induced by AA and/or NEB treatment were quantified by calculating the relative spectra’s peak area ratios normalized by the total AUC of each spectrum. As shown in Table 1, the most prominent changes induced by AA include significant decreases in arginine, glutamate, glutamine and glutathione as well as significant increases in aspartate, glycerophosphocholine and UDP-N-acetylglucosamine. Pretreating cells with NEB slightly inhibited the changes in glutathione and glycerophosphocholine.

**Figure 5:**
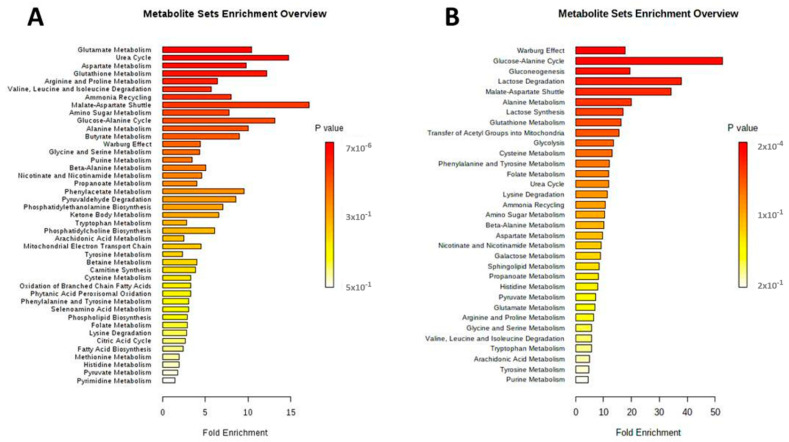
Graphic synopsis of metabolite set enrichment analysis (MSEA) of endothelial cellular extract (**A**) and extracellular supernatant (**B**). Results are expressed in a horizontal bar graph showing the most significant metabolite sets identified during analysis. Bars’ colors are based on *p* values (lower *p* values correspond to darker red), while bars’ lengths are based on the fold enrichment.

Analysis using MSEA showed that the pathways involved in glutamate, aspartate and glutathione metabolism were significantly associated with the effects of AA. The citrulline–arginine cycle and malate–aspartate shuttle were also disrupted by AA (Figure 5A).

### 2.5. Extracellular Metabolic Alterations Induced by AA Exposure

The most relevant extracellular metabolites identified by HMDB are labelled in Table 1. In the cell supernatants, a decrease in lactate and glutamate and an increase in glucose were observed in the AA group compared to controls. The AA-induced decrease in glutamate was significantly inhibited by NEB. MSEA revealed that the most significantly altered metabolic pathways associated with the effects of AA were those involving glycolysis, the glucose–alanine cycle, gluconeogenesis, the metabolism of alanine, glutathione and the malate–aspartate shuttle (Figure 5B).

## 3. Discussion

Although AAs, components of many medicinal plants of the Aristolochia and Asarum species, have been widely demonstrated to be nephrotoxic, mainly for the proximal tubular cells, a growing body of evidence illustrates that capillary rarefaction plays an important role in AA-induced AKI [6,7,24]. Since few studies have investigated AAs’ effects on the endothelial cell, it seemed interesting to evaluate their effects on EAhy926 cells, an endothelial cell line commonly used in studies described in the literature. In the present study, the endothelial cell toxicity of AA was demonstrated by a decreased viability, a change in morphology and the production of ROS. A growing number of studies show that ROS play an important role in AAN [25,26]. Moreover, we previously demonstrated that AA produced oxidative stress in endothelial cells, which may contribute to endothelial dysfunction and cell death [10].

Effective therapeutic strategies to prevent or slow down the renal lesions induced by AA are still lacking. Reducing oxidative stress could constitute a valuable approach. Therefore, we examined the effect of NEB, a beta-blocker known for its antioxidant properties. The results of this study show that pretreatment with NEB significantly restored the cell viability and cell morphology and reduced the ROS production induced by AA in endothelial cells. Interestingly, NEB alone was found to improve endothelial cell viability as compared to the untreated cells. The underlying mechanism should be investigated. The effect of NEB on cell viability was more marked using 10 nM and after 24 h of AA exposure, emphasizing the importance of early treatment. The inhibition of ROS production observed in NEB-pretreated cells could be explained, in part, by the antioxidant capacity of NEB, which has been related to its radical-scavenging activity [27,28] and to its capacity to inhibit NADPH oxidase (NOX) activity [17]. Some studies have suggested that one of the first events leading to the onset and progression of oxidative stress in the vascular system was a decrease in the bioavailability of NO [15]. A growing number of studies have shown that NEB is able to increase eNOS activity following the stimulation of β3-adrenergic receptors [16,18]. According to other authors, NEB is able to reduce the endogenous concentration of asymmetric dimethylarginine (ADMA), an eNOS inhibitor [29]. This increase in eNOS activity induced by NEB could contribute to restoring the bioavailability of NO. Indeed, AA-induced oxidative stress is one mechanism of endothelial dysfunction, as superoxide rapidly inactivates NO and forms peroxynitrite. Consequently, through its antioxidant action and the restoration of NO, NEB could attenuate endothelial dysfunction.

To better understand the mechanism of AA cytotoxicity and the beneficial effect of NEB on endothelial cells, a metabolomic approach was adopted. ^1^H-NMR has been demonstrated to be a valuable approach for studying the oxidative stress response to chemical perturbations [30]. Both intra- and extracellular compartments of EAhy926 cells were analyzed. The baseline experimental conditions (without any toxic agent) of the endothelial cells were first analyzed by examining the extracellular medium. As expected, we observed a consumption of glucose, glutamine and amino acids such as valine, leucine and isoleucine, which represent major substrates for the energy production of the endothelial cell [31,32]. The lactate production observed in our experiments is in agreement with the data of De Bock et al. These authors observed, in human umbilical vein endothelial cells (HUVECs), that the glycolytic pathway was 200 times more active than the glucose oxidation pathway [33], which is consistent with the mitochondrial volume being relatively low in the endothelial cells [34].

After 48 h of cell exposure to AA, analysis of the aqueous intracellular extracts highlighted major metabolic alterations. The most prominent changes included a significant decrease in glutathione, glutamate, glutamine and arginine. The metabolite set enrichment analysis (MSEA) of the endothelial cellular extracts revealed metabolic pathways related to oxidative stress such as glutathione and glutamate metabolism. Glutathione is involved in defending cells against both physiologically and pathologically generated oxidative stress. It is also involved in detoxification processes. Zhang et al. demonstrated that the aristolactam-nitrenium ion intermediate also reacted with glutathione in vitro and in rats, producing phase-two conjugated metabolites [35]. The decrease in glutathione and its precursors glutamate and glutamine in the intracellular compartment could reflect consumption intended either to fight against the oxidative stress induced by AA or to detoxify AA’s metabolites by conjugation reactions.

The NEB pretreatment of AA-exposed cells significantly, but still only partially, restored the glutathione levels, meaning that NEB exerts its antioxidant effects through other mechanisms. In our study, arginine, the substrate for NO, was found to be significantly decreased in cells exposed to AA compared with in the control cells. MSEA of the endothelial cellular extract revealed that aspects of the urea cycle corresponding to the citrulline–arginine cycle in the endothelial cells and arginine/proline metabolism were perturbed. The involvement of the citrulline–arginine cycle in the restoration of arginine levels in endothelial cells was described by Hecker et al. and facilitates optimal NO signaling [36,37]. Our experiments showed that NEB was able to inhibit the decrease in arginine due to AA exposure, leading to enhanced eNOS activity and contributing, therefore, to restoring NO bioavailability. Moreover, Mihout et al. suggested that ADMA, known to inhibit eNOS by competing with arginine, contributes to endothelial dysfunction during the development of renal fibrosis [38]. The ability of NEB to reduce the endogenous concentration of ADMA could explain the restoration of NO bioavailability [29].

We also observed, in our experiments, an increase in aspartate. Aspartate is generally catabolized into intermediates that enter the TCA cycle, reflecting its lower consumption as a glucogenic amino acid during AA exposure. This observation clearly indicates an alteration or dysfunction of the mitochondria induced by AA. Similar findings supporting the hypothesis of Krebs cycle disruption by AA have been reported in other studies [21,39]. MSEA demonstrated dysfunction of the malate–aspartate shuttle. The malate–aspartate shuttle translocates reducing equivalents from the cytoplasm to mitochondria. The changes in the levels of glutamate observed in our study could inhibit the malate–aspartate shuttle and oxidative phosphorylation [40]. An increase in glucosamine levels, and especially UDP-N-acetyl-glucosamine, was also observed. UDP-N-acetyl-glucosamine is the end-product of the hexosamine metabolic pathway produced from glucose derivatives. This metabolic pathway is closely related to glycolysis. UDP-N-acetyl-glucosamine is involved in glycosylation processes. The alteration of this mechanism can lead to an accumulation of malformed proteins, endoplasmic reticulum stress, and, in severe cases, an apoptotic response. In our experiments, it seemed that the hexosamine pathway was abnormally activated by AA, as the levels of UDP-N-acetyl-glucosamine increased while those of glutamine decreased. Some authors have observed that glycosylation protects proteins against free radicals generated from toxic xenobiotics [41]. From this, it would be tempting to argue that the activation of glycosylation is part of a defense mechanism protecting against the free radicals induced by AA. Our approach also revealed a significant increase in glycerophosphocholine. This result is consistent with previous studies that showed an increase in glycerophosphocholine in the renal tissue of AA-treated rats [42,43]. Glycerophosphocholine plays an important role in the structural integrity of the cell membrane [44]. Some authors suggest that the increase in glycerophosphocholine could be a mechanism protecting against the cell damage induced by oxidative stress [45,46]. Furthermore, in the presence of NEB, known for its antioxidant properties, a lower increase in glycerophosphocholine was observed.

The metabolomic analysis of extracellular media can provide information on the metabolites released by the cells and on the substances contained in the culture medium that have not been consumed by the cells. In our experiments, the analysis of cell culture supernatants showed a decrease in lactate and glutamate as well as an increase in glucose in the extracellular medium of cells exposed to AA, indicating that both glycolytic metabolism and mitochondrial glucose oxidation were downregulated during AA exposure. Several authors have shown mitochondrial dysfunction in AA-related nephrotoxicity [26,47]. The subsequent alteration of the Krebs cycle could explain the decrease in glucose consumption and, hence, greater concentration of this metabolite in the extracellular medium [48]. In addition, a slowdown in lactate production should not be ruled out given the decreased lactate content in the extracellular environment of the cells exposed to AA. The increased activity of the hexosamine biosynthetic pathway observed in the AA-exposed cells could participate in the reduction of lactate excretion. The culture medium contains glutamine and is devoid of glutamate. Therefore, it is most likely that the glutamate observed in the medium comes from an efflux of glutamate through a transporter. The cystine/glutamate transporter allows the uptake of cystine via exchange with glutamate across the cell membrane for glutathione (GSH) biosynthesis to alleviate oxidative stress [49]. In our experiments, a decreased glutamate level was observed in the extracellular medium from cells incubated with AA. High glutamate export in the oxidative condition could result in a partial depletion of intracellular glutamate, leading to a decreased efflux of glutamate [50]. The decreased glutamate observed in the intracellular compartment, probably due to increased synthesis of glutathione, either to fight against oxidative stress or to detoxify the metabolites of AA by conjugation reactions, could also explain the reduced glutamate efflux.

## 4. Conclusions

**Figure 6:**
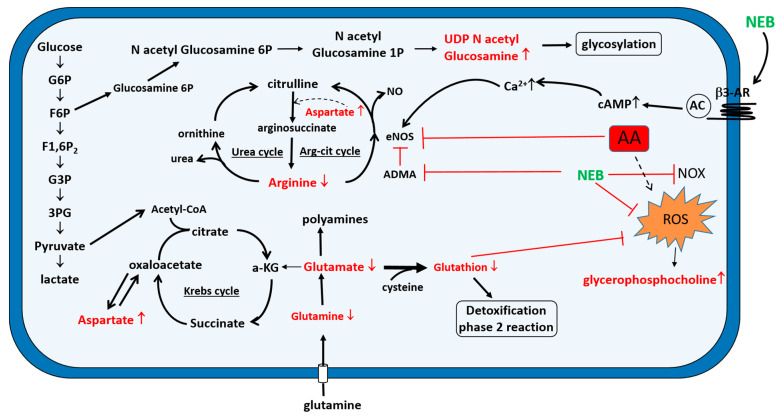
Proposed metabolic pathway affected by AA and the protective effects of NEB. AAs increase the production of ROS, which leads the cell to fight oxidative stress (1) through glutathione and its precursors (glutamate and glutamine) and (2) by promoting glycosylation that protects proteins against free radicals. AAs decrease the expression of eNOS and the bioavailability of NO. AAs affect the Krebs cycle and citrulline–arginine (cit–arg) cycle, with decreased arginine production and increased aspartate. NEB decreases ROS (1) by the scavenger effect and (2) by activating eNOS following its binding to the β3 adrenergic receptor (AR) and inhibiting ADMA, which leads to an increase in the bioavailability of NO. ↑ = increase; ↓ = decrease; ⊥ = inhibition; → = activation.

We provide evidence that the exposure of endothelial cells to AA results in cytotoxicity associated with oxidative stress and metabolic alterations that could lead to cell death. Our metabolomic analyses highlight AA-induced changes in metabolites involved in metabolic pathways such as the glutamate, glutathione aspartate, arginine metabolism, citrulline–arginine and Krebs cycles. As shown in Figure 6, all these results provide a preliminary demonstration of AA-induced alterations, consistent not only with oxidative stress and mitochondrial dysfunction leading to endothelial damage but also with defense mechanisms protecting against free radicals. The reduction of oxidative stress could be one of the ways to mitigate the toxic effects of AA in endothelial cells and the subsequent disappearance of microvasculature. The antioxidant properties of NEB could partly explain its cytoprotective effects on endothelial cells damaged by AA.

## 5. Materials and Methods

### 5.1. Chemicals

A mixture of AAI and II (50% of each compound) was purchased from Acros Organics Co. (Geel, Belgium). Dulbecco’s Modified Eagle Medium (DMEM), penicillin/streptomycin, L-glutamine and accutase were obtained from Capricorn Scientific (Ebsdorfergrund, Germany). Fetal bovine serum (FBS) was purchased from Life Technologies Europe (Gent, Belgium), and sodium citrate, from Merck (Darmstadt, Germany). Resazurin, 2′,7′-dichlorodihydrofluorescein diacetate (DCFDA) and nebivolol hydrochloride were obtained from Sigma (St. Louis, MO, USA).

### 5.2. Cell Cultures

The human endothelial cell line EAhy926 was purchased from the American Type Culture Collection (ATCC, Molsheim, France), and the cells were grown in a DMEM high-glucose medium supplemented with 10% FBS and 1% penicillin/streptomycin at 37 °C under 5% CO_2_. When they had reached about 80–90% confluence, the cells were harvested and seeded on 96-well plates (1 × 10^4^ cells), six-well plates (4 × 10^5^ cells) and a 75 cm^2^ flask (5 × 10^6^ cells) from Greiner bio-One (Vilvoorde, Belgium). The cells were then incubated in complete medium for 24 h and treated with 50 µM AA and/or different doses of NEB (5, 10, 15, 20 nM) in FBS-depleted low-glucose DMEM for 24 h or 48 h.

### 5.3. Cell Viability

The cytotoxicity of the tested samples was assessed by the resazurin reduction assay. This assay is based on the reduction of the indicator dye, resazurin, to the highly fluorescent resorufin by viable cells. Cells were treated with 50 µM AA and different doses of NEB (5, 10, 15, 20 nM) in 96-well plates. After 24 or 48 h, the cells were washed twice with PBS and incubated with 0.44 mM resazurin solution at 37 °C for 2 h. The absorbance at 540 and 620 nm wavelengths was measured using an iEMS Reader MF spectrophotometer (Thermo Labsystems, Breda, The Netherlands). Each assay was performed at least three times, with six replicates each.

### 5.4. Microscopic Examination

To evaluate the impact of AA on the morphology, EAhy926 cells were incubated with 50 µM AA and/or 10 nM NEB for 24 h and 48 h in six-well plates. The cells were imaged using a Motic AE21 phase-contrast microscope equipped with a Moticam 2300 camera (Motic, Wetzlar, Germany) at a magnification of 200×.

### 5.5. Measurement of ROS

Following 24 h of treatment in six-well plates, cells were equilibrated for 10 min in HBSS at 37 °C and treated with 5 µM DCFDA solution at 37 °C for 15 min. Afterward, the cells were harvested with accutase, centrifuged and analyzed by flow cytometry (FACS Canto II, BD Biosciences, San Jose, CA, USA) for ROS detection. The DCFDA fluorescence was measured at an excitation wavelength of 488 nm and an emission wavelength of 535 nm (FL-1). A total of 10^4^ cells were recorded, and the mean fluorescence intensities were estimated and are expressed as percentages of the untreated control.

### 5.6. ^1^H-NMR Samples’ Collection and Extraction

The 48 h culture media were transferred from 75 cm^2^ tissue culture flasks into sterile 15 mL conical tubes and centrifuged at 1500× *g* for 5 min at room temperature to pellet any cellular debris. The remaining cells were rinsed twice with 7 mL of ice-cold phosphate-buffered saline (pH 7.4). The cells were quenched using 3 mL of ice-cold methanol to terminate metabolic reactions and then harvested by scraping. The cell suspension was then mixed with methanol:water:chloroform (1:0.9:1) to separate the aqueous phase from the organic phase. The supernatant was transferred to fresh tubes, dried and stored at −80 °C until further evaluation. The polar phase containing the intracellular metabolite mixture was dried using a SpeedVacuum and stored at −80 °C before analysis.

### 5.7. ^1^H-NMR Spectroscopy

The dried residues were reconstituted with 700 μL of phosphate buffer prepared in a mixture of H_2_O/D_2_O (80:20; *v*:*v*). Following vortexing and centrifugation at 13,000× *g* for 10 min, 650 μL of supernatant was mixed with 50 µL of a 7 mM solution of 3-trimethylsilyl propionic-2,2,3,3-d4 acid (TSP) prepared in 100% deuterium oxide and transferred into 5 mm NMR tubes for NMR analyses. The acquisition of the ^1^H-NMR spectra was performed on a Bruker 600-MHz NMR spectrometer (Bruker Avance, Germany) using the NOESYPRESAT-1D pulse sequence, 128 scans, 32,768 data points, a spectral width of 6410.2 Hz, an acquisition time of 2.5 s and a pulse recycle delay of 2 s.

### 5.8. Multivariate Data Analyses and Metabolite Identification

The MestRe Nova 10 software (Mestre Lab Research, Santiago de Compostela, Spain) was used for the phase and baseline corrections of all the spectra. The chemical shift was referenced to TSP and binned in subregions of 0.04 ppm sections. The spectral binned data were converted to the Microsoft Excel format, and the AUC of one isolated chemical shift, previously normalized using the total AUC of each spectrum, was calculated. The data were imported into the SIMCA-P+ 12.0 software (Umetrics, Umeå, Sweden) for multivariate statistical analysis. Principal component analysis (PCA) followed by a partial least-square discriminant analysis (PLS-DA) was performed to examine the intrinsic variation in the dataset. PCA was used to highlight the possible group separation observed in the score plot and to identify potential outliers. PLS-DA was used to identify significant discriminant descriptors (metabolites). These metabolites were ranked according to their variable influence on projection (VIP) scores. When VIP > 1, the variables were considered statistically significant variables and their corresponding metabolites were identified. Metabolites were identified using the Chenomx NMR suite software (version 8.1.1) (Edmonton, Canada) and the human metabolome database (HMDB). To highlight the biological pathways possibly linked to the discriminant metabolites, the MetaboAnalist 4.0 online software for metabolite set enrichment analysis (MSEA) was used.

### 5.9. Statistical Analyses

All the data for cell viability and oxidative stress are expressed as the means ± SEMs. Differences between cell culture conditions were evaluated by analysis of variance (ANOVA) followed by a post hoc analysis (Bonferroni’s multiple-comparison test) using Prism 5 (GraphPad Software, Inc., San Diego, CA, USA). *p* < 0.05 was considered significant. Regarding the metabolomics, statistical significance was determined by integrating the NMR peaks of each of the metabolites that contributed to the multivariate separation using an ANOVA followed by Sidak’ multiple comparisons test, and *p* < 0.05 was considered significant.

## Figures and Tables

**Table 1:** Identified discriminant metabolites with corresponding chemical shifts for 48 h exposure. Note: Metabolite AUC changes are indicated by arrows. Data are expressed as mean ± SEM of at least six independent experiments. Statistical analysis was performed by one-way ANOVA followed by Sidak’s multiple comparisons test. * *p* < 0.05, ** *p* < 0.01, *** *p* < 0.001 and **** *p* < 0.0001 vs. control ^#^ *p* < 0.05 and ^##^ *p* < 0.01 vs. AA.

Metabolites	Chemical Shift (ppm)	AA vs. Ctrl	AA + NEB vs. AA
**1. Intracellular Extracts**			
Arginine	3.76	↓ ****	-
Aspartate	2.82	↑ *	-
Glutamate	2.32	↓ *	-
Glutamine	2.44	↓ *	-
Glutathione	2.54	↓ ****	↑ ^#^
Glycerophosphocholine	3.24	↑ *	↓ ^#^
UDP-N-acetylglucosamine	5.96	↑ **	-
**2. Extracellular Fluids**			
Glucose	3.4	↑ *	-
Glutamate	2.32	↓ ***	↑ ^##^
Lactate	1.32	↓ ***	-

## Data Availability

Not applicable.

## Abbreviations

AAs: Aristolochic acids; AAN: aristolochic acid nephropathy; ADMA: asymmetric dimethylarginine; AKI: acute kidney injury; AR: adrenergic receptor; Arg: arginine; Asp: aspartate; CKD: chronic kidney disease; DCFDA: 2′,7′-dichlorodihydrofluorescein diacetate; eNOS: endothelial nitric oxide synthase; Glc: glucose; Gln: glutamine; Glu: glutamate; GPC: glycerophosphocholine; GSH: glutathione; HMDB: Human Metabolome Database; ^1^H NMR: proton nuclear magnetic resonance; HUVECs: human umbilical vein endothelial cells; Ile: isoleucine; Lac: lactate; Leu: leucine; MSEA: metabolite set enrichment analysis; NEB: nebivolol; nm: nanometer; NO: nitric oxide; PCA: principal component analysis; PLS-DA: partial least squares discriminant analysis; ROS: reactive oxygen species; TCA cycle: tricarboxylic acid cycle, also called Krebs cycle; UDP-GlcNAC: UDP-N-acetylglucosamine; Val: valine; VIP: variable influence on projection.
